# Synthesis of Ag@Pd Nanocubes and Pd‐based Nanoframes via One‐Shot Injection of a Halide‐Free Precursor for Continuous Production in a Flow Reactor

**DOI:** 10.1002/chem.202500201

**Published:** 2025-03-21

**Authors:** Hansong Yu, Jianlong He, Kei Kwan Li, Qijia Huang, Yong Ding, Younan Xia

**Affiliations:** ^1^ School of Materials Science and Engineering Georgia Institute of Technology, Atlanta Georgia 30332 United States; ^2^ School of Chemistry and Biochemistry Georgia Institute of Technology, Atlanta Georgia 30332 United States; ^3^ The Wallace H. Coulter Department of Biomedical Engineering Georgia Institute of Technology and Emory University, Atlanta Georgia 30332 United States

**Keywords:** nanocrystals, nanoframes, continuous flow, galvanic replacement, reduction kinetics

## Abstract

Noble‐metal open nanostructures have remarkable catalytic capabilities toward a wide range of reactions. In particular, Pd‐based open nanostructures have been synthesized and validated for superior catalytic performance toward formic acid oxidation. However, most of the syntheses are based on dropwise addition, making it challenging to increase the production volume. In this work, we present a facile approach to the synthesis of Ag@Pd core‐frame nanocubes and then Pd‐based nanoframes through one‐shot injection. In a typical synthesis, Ag nanocubes are dispersed in an aqueous solution of ascorbic acid and polyvinylpyrrolidone at room temperature, followed by the injection of Pd(NH_3_)_4_(NO_3_)_2_ precursor in one shot. The Pd(NH_3_)_4_(NO_3_)_2_ precursor has a much slower reduction kinetics relative to Na_2_PdCl_4_, preventing self‐nucleation and enabling controlled deposition of Pd atoms on the Ag nanocubes. The lower reduction potential of Pd(NH_3_)_4_(NO_3_)_2_ also helps minimize the galvanic replacement reaction, facilitating uniform deposition of Pd atoms. After selectively etching away the Ag template, Pd‐based nanoframes with a rigid cubic structure are obtained. Finally, the synthesis is successfully adapted to a continuous flow system, generating Ag@Pd nanocubes with comparable quality to those obtained *via* one‐shot synthesis, demonstrating a practical route to large‐scale production of Pd‐based nanoframes with H_2_O_2_ etching.

## Graphical abstract

We report the synthesis of Pd‐based nanoframes by templating with Ag nanocubes *via* one‐shot injection of Pd(NH_3_)_4_(NO_3_)_2_ solution and H_2_O_2_ etching. We also extend the one‐shot synthesis from a batch to a continuous flow reactor for continuous production without compromising the quality.

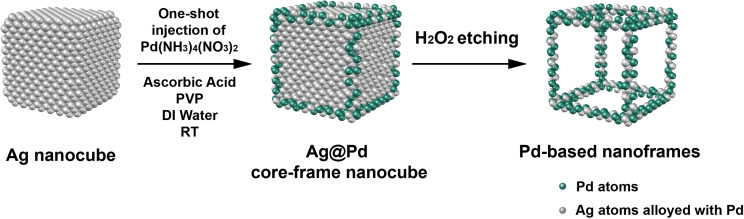

## Introduction

Noble‐metal nanocrystals featuring unique shapes and/or structures have received great attention for a variety of applications, including those in catalysis,[^1^, ^2^, ^3^] photonics,[^4^, ^5^, ^6^] and biomedicine.[^7^, ^8^, ^9^] Specifically, Ag‐derived bimetallic nanocrystals and their derivatives with an open structure (*e. g*., nanoframes and nanocages) have been extensively investigated due to their remarkable optical and catalytic properties.[^10^, ^11^, ^12^, ^13^, ^14^] For instance, the Pt‐based nanocages derived from Ag nanocubes showed enhanced performance toward the oxygen reduction reaction over a commercial Pt/C.^[15]^ The Ru‐based nanocages derived from Ag nanocubes exhibited superior performance in the reduction of 4‐nitrophenol by NaBH_4_.^[16]^ Most of the intermediate, core‐shell or core‐frame, nanostructures were prepared through dropwise addition of the metal precursor. By adding the precursor in a dropwise fashion, steady‐state reduction kinetics could be achieved, allowing for slow but well‐controlled deposition of the second metal on the template while eliminating self‐nucleation.[^17^, ^18^]

The reduction rate of a synthesis is directly proportional to the concentrations of both the reducing agent and precursor. In the case of a synthesis involving an excessive amount of reducing agent, the reduction rate can be approximated as a pseudo‐first‐order reaction with respect to the precursor concentration:

(1) $$R=-\frac{d\left[M^{n+}\right]}{dt}=k\times \left[M^{n+}\right]$$

where R is the reduction rate, k is the rate constant, and $\left[M^{n+}\right]$ is the molar concentration of the precursor. After integrating eq. (1), we obtain the reduction rate for one‐shot injection as:

(2) $$R=k{\left[M^{n+}\right]}_{0}\times e^{-kt}$$

For a synthesis involving one‐shot injection, the precursor concentration and thus the reduction rate would decay exponentially, making it challenging to achieve a steady‐state synthesis. This challenge can be addressed by switching to dropwise addition of the precursor solution. In this case, the precursor concentration in the reaction mixture and thus the reduction rate would only oscillate within a narrow range while staying at a relatively low level,[^17^, ^18^] leading to a pseudo steady‐state synthesis. However, due to the limited size of each droplet and the long time it takes to introduce all the precursor, the dropwise method is not well‐suited for scaling up the production of nanocrystals. In comparison, one‐shot injection is more viable for large‐scale synthesis due to its short reaction time and great potential for use with a continuous flow reactor.[^19^, ^20^, ^21^] However, the rapid reduction at the beginning of a one‐shot synthesis can lead to self‐nucleation and thereby the formation of undesired products. To address this issue, it is critical to achieve a slow, steady‐state reduction rate similar to the case of dropwise synthesis but using one‐shot injection. As shown in eq. (2), this goal can be realized by switching to a precursor with a very small rate constant k . In this case, the reduction rate still follows an exponential decay over time but the overall change is so small that the reduction rate can be approximated as a steady state.

Nanostructures based on Pd are well recognized as excellent catalysts for a broad range of electrocatalytic reactions, particularly, the formic acid oxidation (FAO) key to the operation of direct formic acid fuel cells.^[22]^ Most of the successful syntheses of Pd nanocrystals reported in the literature involved the reduction of Na_2_PdCl_4_ or Na_2_PdBr_4_ in an aqueous or polyol system.^[23]^ It is well‐documented that PdBr_4_^2−^ has a slower reduction kinetics relative to PdCl_4_^2−^, meaning that the former has a smaller rate constant *k*. As such, Br^−^ ions are often added into a synthesis to help slow down the reduction kinetics and help prevent self‐nucleation.^[24]^ However, the presence of Br^−^ ions also increases the complexity of a synthesis due to the strong binding Br^−^ to Pd(100) surface and the possible initiation of oxidative etching.[^25^, ^26^] Therefore, it would be advantageous by working with halide‐free precursors, such as those involving a ligand such as amine or organic compound that binds strongly to Pd^2+^ ions. In addition to its effect on the rate constant, the strong binding between the ligand and Pd^2+^ can also significantly influence the reduction potential of the precursor to potentially alleviate the galvanic replacement reaction (GRR).[^27^, ^28^] While GRR offers a simple method for synthesizing bimetallic nanocrystals, it often leads to fast and non‐uniform deposition due to the dissolution of the template and subsequent alternation to the morphology.^[29]^ Especially for the synthesis of Pd‐based nanoframes by templating with Ag nanocubes, GRR can quickly occur between the Pd precursor and Ag template, as driven by the low reduction potential of Ag and the positive reaction potential of the overall reaction.[^30^, ^31^] By switching to a Pd precursor containing a strong ligand and thus a low reduction potential, it is feasible to reduce the driving force for GRR and even make the reaction unspontaneous. We argue that the use of a precursor containing a strong binding ligand may also allow for the production of hollow nanostructures in the one‐shot setting, without compromising the control and quality.

Once demonstrated for one‐shot synthesis in a batch reactor, we can immediately extend the protocol to a continuous flow reactor for large‐scale synthesis.^[32]^ The flow reactor typically consists of a fluidic tube or channel, together with connectors and mixers. When the reagents are introduced into the fluidic tube, they will be mixed, and heated if necessary, to initiate the reduction reaction while flowing through the reactor continuously. This setting is more or less identical to that of a conventional one‐shot synthesis involving a batch reactor, where all the reagents are directly added and mixed at the beginning of a synthesis. However, the use of a continuous flow rather than batch reactor offers the immediate advantage for large‐scale production as it has been demonstrated for a number of metals and their alloys.[^19^, ^20^, ^33^, ^34^]

Herein, we report the successful synthesis of Ag@Pd nanocubes by one‐shot injection of Pd(NH_3_)_4_(NO_3_)_2_ precursor into an aqueous suspension of Ag nanocubes in the presence of ascorbic acid (AA) and poly(vinylpyrrolidone) (PVP) at room temperature. We then obtain Pd‐based nanoframes by selectively etching away the Ag template with aqueous H_2_O_2_. As a new development, Pd(NH_3_)_4_(NO_3_)_2_ was confirmed with a slower reduction kinetics, enabling the one‐shot injection of the Pd precursor without invoking self‐nucleation alongside the deposition of Pd atoms on Ag nanocubes. In addition, due to its lower reduction potential relative to Na_2_PdCl_4_, the GRR between Ag and Pd precursor is minimized, leading to the uniform deposition of Pd atoms on Ag nanocubes. By gradually increasing the total volume of the precursor introduced into the reaction suspension, we can control the growth pattern of the Pd atoms to have them preferentially deposited on the edges and corners of the Ag nanocubes prior to diffusion to the side faces. We also investigated the deposition of Pd atoms over time, further revealing the growth pathway. Finally, we extend the one‐shot protocol from a batch to a continuous flow reactor, and successfully synthesize Ag@Pd nanocubes and then Pd‐based nanoframes of comparable quality to those produced in one‐shot injection synthesis, validating the potential for large‐scale production.

## Results and Discussion

**Figure 1:**
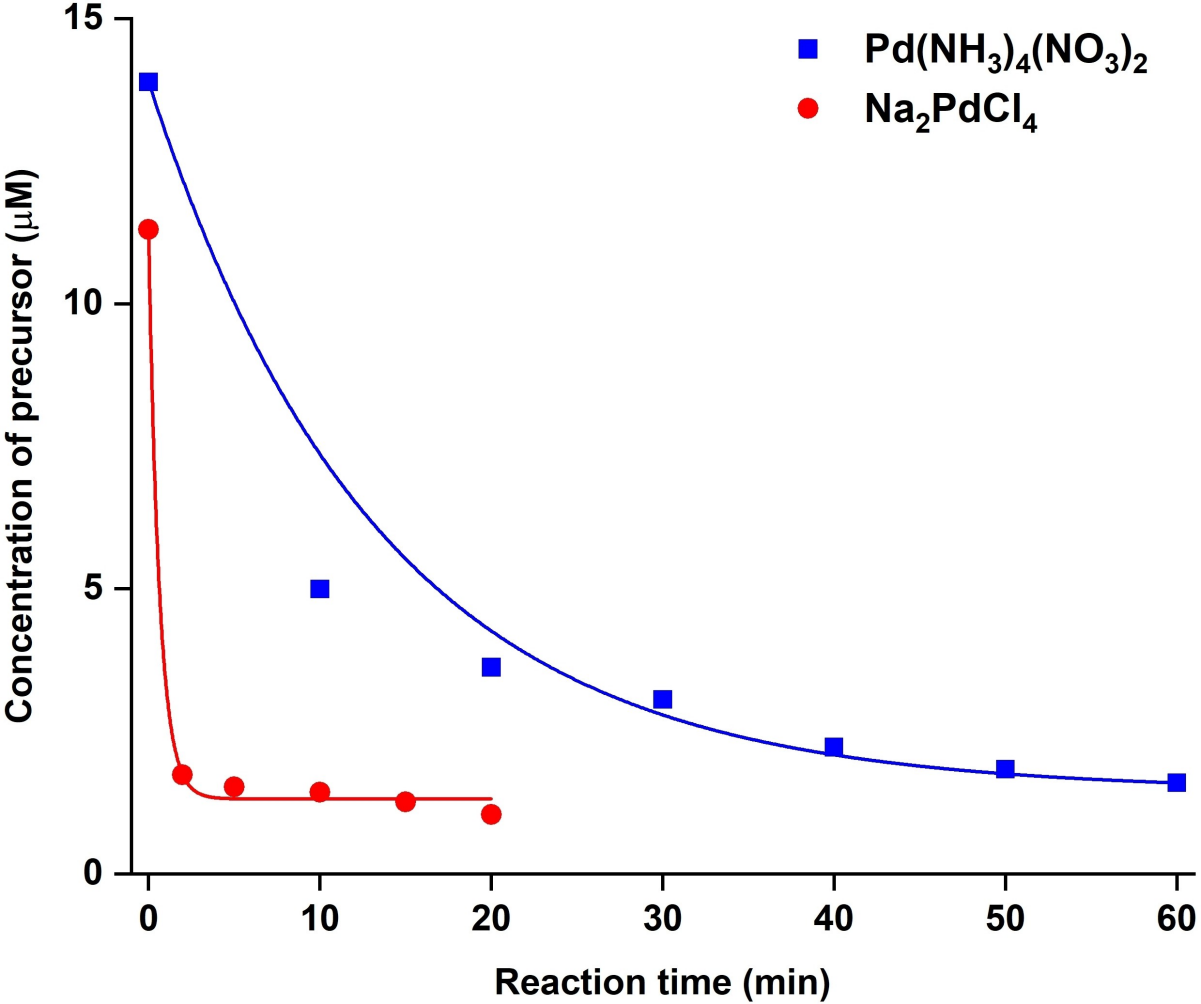
Plots of the concentrations of the remaining precursor in the reaction mixture as a function of time for one‐shot synthesis involving Pd(NH_3_)_4_(NO_3_)_2_ and Na_2_PdCl_4_, respectively.

We followed the published protocol to synthesize Ag nanocubes with an average edge length of about 35 nm (Figure S1).^[35]^ For the first set of experiments, we analyzed the reduction kinetics to validate our hypothesis that the Pd(NH_3_)_4_(NO_3_)_2_ precursor had a slower reduction rate compared to the conventional precursor, Na_2_PdCl_4_. In a typical study, 0.2 mL of each precursor was injected into a mixture of Ag nanocubes, PVP, and AA in one shot. The concentrations of the remaining Pd(II) in the reaction mixture were measured by sampling aliquots at various time points and then analyzing the Pd contents in the supernatants using inductively‐coupled plasma mass spectrometry (ICP‐MS). As shown in Figure 1, the concentration of the remaining Pd(II) had a much slower decay for Pd(NH_3_)_4_(NO_3_)_2_ relative to that of Na_2_PdCl_4_, indicating a slower reduction rate for the former precursor. In addition, the data points for the Pd(NH_3_)_4_(NO_3_)_2_ system gave a better exponential curve fitting as a result of the pseudo‐first‐order kinetics associated with the chemical reduction. In comparison, the data points for the Na_2_PdCl_4_ system gave a poor exponential fitting due to the mixing of chemical reduction and galvanic replacement. The combined consumption of Na_2_PdCl_4_ resulted in a steep drop in precursor concentration within the first 5 min of the synthesis. The fast kinetics resulted in not only Pd‐based nanoframes with reduced uniformity and cubic conformity but also significant homogeneous nucleation as the excessive amount of Pd atoms generated within a short period of time led to supersaturation in the reaction mixture. Significantly, Pd(NH_3_)_4_(NO_3_)_2_ could mitigate these two issues by rendering a slower reduction kinetics and minimal involvement of galvanic replacement, making it a desired precursor for the one‐shot synthesis of Ag@Pd core‐frame nanocubes.

**Figure 2:**
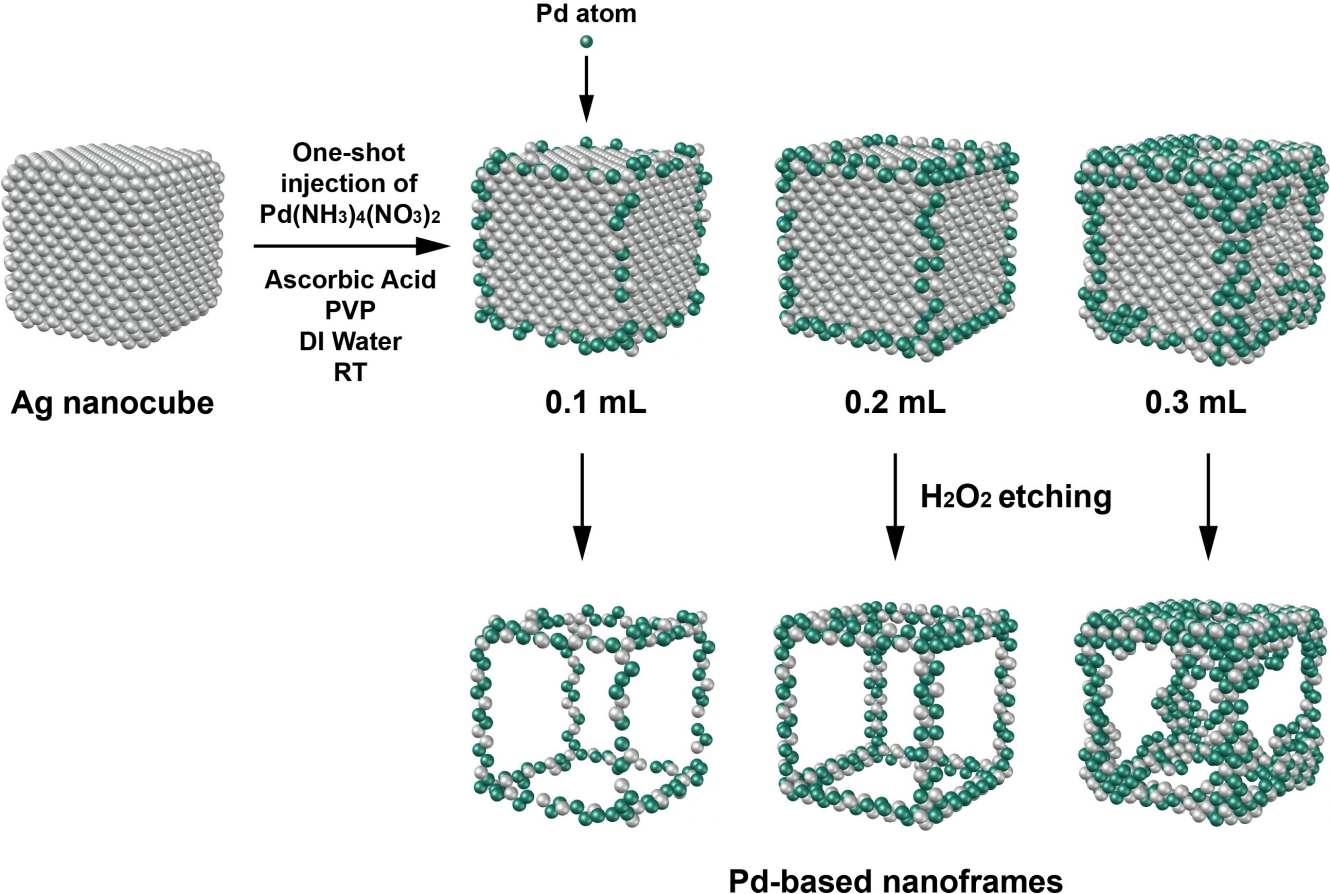
Schematic illustration showing the transformation of Ag nanocubes into Pd‐based nanoframes through the reduction of Pd(NH_3_)_4_(NO_3_)_2_ precursor for the deposition of Pd atoms, followed by selective etching of the Ag core.

After confirming the slower reduction kinetics of Pd(NH_3_)_4_(NO_3_)_2_, we investigated the deposition of Pd atoms on the surface of Ag nanocubes with different volumes of Pd(NH_3_)_4_(NO_3_)_2_ being added in one shot. In a typical synthesis, we dispersed the Ag nanocubes in an aqueous mixture of PVP and AA under magnetic stirring, followed by one‐shot injection of aqueous Pd(NH_3_)_4_(NO_3_)_2_ at room temperature. In this case, Pd(NH_3_)_4_^2+^ was reduced by AA to Pd atoms for their preferential deposition onto the edges and corners of the Ag nanocubes. During the deposition, the Pd and Ag atoms also inter‐diffuse to form a Pd−Ag alloy. Afterwards, Pd‐based nanoframes were obtained by selectively etching away the Ag core using H_2_O_2_ (Figure 2).

**Figure 3:**
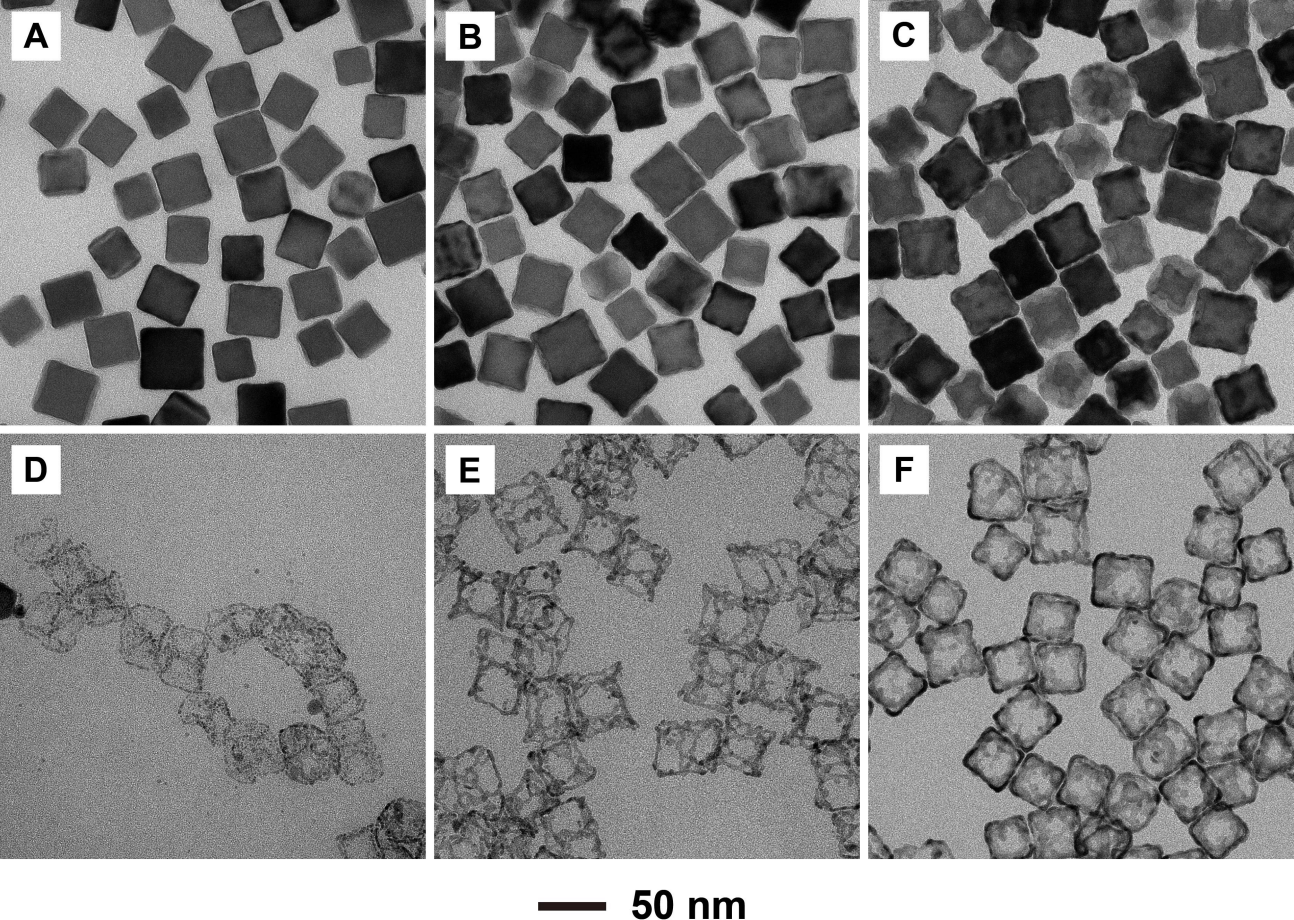
TEM images of the Ag@Pd nanocubes (A–C) before and (D–F) after etching with 3 % aqueous H_2_O_2_. The samples were prepared by one‐shot injection of different volumes of aqueous Pd(NH_3_)_4_(NO_3_)_2_: (A and D) 0.1, (B and E) 0.2, and (C and F) 0.3 mL.

Figure 3A–C shows TEM images of the Ag@Pd nanocubes prepared with the injection of 0.1, 0.2, and 0.3 mL of Pd(NH_3_)_4_(NO_3_)_2_ solution, respectively. In the case of 0.1 mL of precursor, the products showed sharper corners and rougher edges when compared to the Ag nanocubes. This indicated that Pd atoms were deposited preferentially on the corners and edges at a small volume of the precursor. In principle, the surface energies of the different facets of Ag nanocubes increase in the order of γ_111_ < γ_100_ < γ_110_.^[36]^ With PVP as a capping agent to selectively passivate the {100} facets, the surface energy of {100} facets would be reduced, resulting in a new order of γ_100_ < γ_111_ < γ_110_.^[37]^ Therefore, the newly‐formed Pd atoms were preferentially deposited on {110} and {111} facets, which corresponds to the edges and corners of nanocubes. With more Pd precursor being injected, the edges became rougher as more Pd atoms were deposited on these sites, implying the involvement of island growth rather than layer‐by‐layer growth. This trend could be attributed to the relatively large (*ca*. 5.1 %) lattice mismatch between Ag(110) and Pd(110) and the involvement of room temperature. With the injection of 0.3 mL of precursor, the Ag nanocubes showed a gradient in contrast on the side faces in addition to the more deposition of Pd atoms on the edges, indicating the deposition of some Pd atoms on the {100} facets as well.

To further reveal the locations of Pd deposition while obtaining Pd‐based nanoframes, the Ag core was selectively etched away using aqueous H_2_O_2_. As shown in Figure 3D, Pd‐based nanoframes with extremely thin ridges were obtained by etching the Ag@Pd sample prepared with 0.1 mL of the precursor. Due to the limited supply of Pd atoms from the precursor, small Pd−Ag clusters with a size of around 3 nm were formed and connected loosely with each other to form the Pd‐based nanoframes. With the injection of 0.2 mL of the precursor, more Pd atoms were formed and deposited, and evenly distributed along the edges, generating rigid Pd‐based nanoframes. Compared to the deformed Pd‐based nanoframes prepared with 0.1 mL of the precursor, the nanoframes obtained with 0.2 mL of the precursor were rigid enough to resist the capillary force during the evaporation of water when the sample was dried for TEM characterization, revealing a cubic frame structure (Figure 3E). Finally, with the injection of 0.3 mL of the precursor, the resultant nanoframes exhibited a well‐defined cubic shape inherited from the Ag nanocube template, with more Pd atoms growing on both the edges and side faces (Figure 3F). The Pd atoms deposited on the side faces largely came from the surface diffusion of Pd adatoms from the edges.^[38]^

To further elucidate the crystal structure of the Pd‐based nanoframe, we collected a powder X‐ray diffraction (XRD) pattern from the sample associated with Figure 3F. As shown in Figure S2, the red and blue dashed lines correspond to the characteristic peaks of Ag (JCPDS No. 04‐0783) and Pd (JCPDS No. 46‐1043), respectively. The peaks appear between those of Ag and Pd, indicating the formation of a Pd−Ag alloy in the nanoframes. The energy‐dispersive spectroscopy (EDS) analysis also confirms the formation of a Pd−Ag alloy (Figure S3). According to the peak areas in the spectrum, the alloy atomic composition was *ca*. 30 % Pd and 70 % Ag, indicating the formation of Pd_1_Ag_2_ alloy. It should be pointed out that Pd−Ag alloy nanostructures with a similar composition have been reported to exhibit excellent catalytic performance in various reactions, such as hydrogen production from formic acid and CO_2_ hydrogenation to generate formic acid.[^39^, ^40^] This highlights the potential catalytic capabilities of the Pd‐based nanoframes.

**Figure 4:**
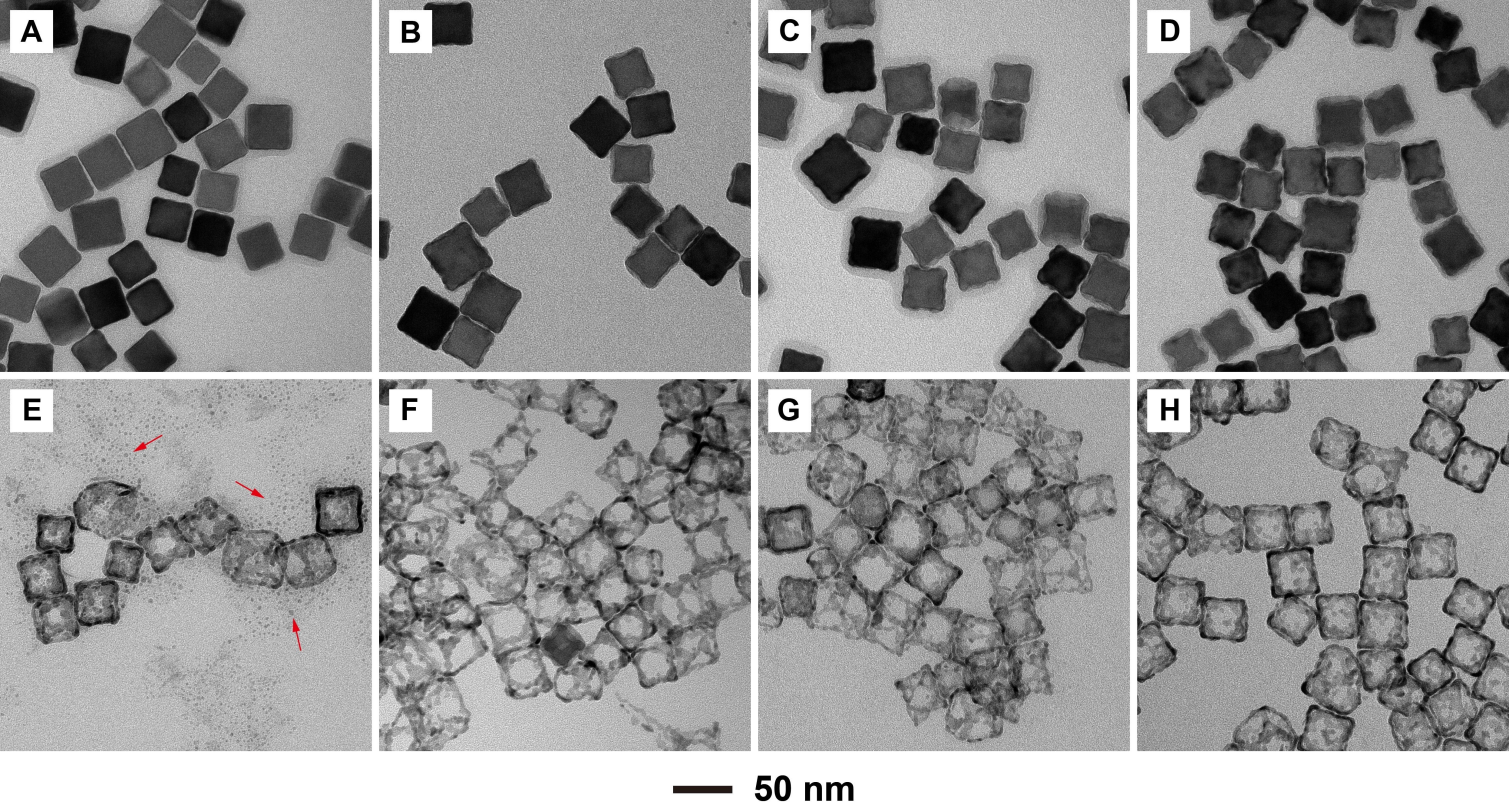
TEM images of the Ag@Pd core‐frame nanocubes prepared with the use of 0.3 mL of Pd(NH_3_)_4_(NO_3_)_2_ at different time points: (A) 15, (B) 30, (C) 45, and (D) 60 min, and (E–H) their resultant nanoframes after etching the samples with aqueous H_2_O_2_. The arrows in (E) mark the 3 nm Pd particles coexisting with the nanoframes.

We also investigated the growth pattern of Pd atoms on Ag nanocubes in a time‐elapsed study. During a standard synthesis with 0.3 mL of the precursor, aliquots were sampled from the reaction mixture at different time points and etched with H_2_O_2_ to better observe the deposition of Pd atoms. At *t*=15 min into the synthesis, small islands of Pd were observed on the edges of the nanocubes, as shown in Figure 4A. Figure 4B–D shows further deposition of Pd atoms on the nanocubes. The edges became increasingly rough with reaction time, indicating more deposition of Pd atoms. The deposition of Pd islands was further confirmed by imaging the sample after Ag etching, where a large amount of Pd nanoparticles with a size of around 3 nm were found (Figure 4E). These Pd particles offer direct evidence to support the island growth of Pd atoms on Ag nanocubes. The individual Pd islands were not fused together at the beginning of the synthesis and thus fell apart during the etching of Ag template. From the corresponding nanoframes, we also noticed an increase in thickness for the ridges of the nanoframes and the coverage of the side faces by Pd atoms (Figure 4, F–H). It is worth mentioning that at *t*=30 min into the synthesis, the Pd‐based nanoframes were already rigid enough to sustain the cubic shape after the removal of the Ag template. After another 15 or 30 min, only minimal growth was observed at the edges, with increased deposition on the side faces in the final product, suggesting that deposition slowed over time and surface diffusion became prevalent. This trend is due to the nature of one‐shot injection that gives a high concentration of precursor at the beginning and thus fast reduction and consumption of the precursor. Typically, both the precursor concentration and the accompanied reduction rate experienced an exponential decay as a function of time.^[17]^

In addition to precursor volume and reaction time, the influence of reduction kinetics on the deposition of Pd atoms on Ag nanocubes was also investigated using different precursors. In a control experiment, we substituted the new precursor with the conventional Na_2_PdCl_4_ precursor in a standard one‐shot injection synthesis, which possessed a much faster reduction kinetics as shown in Figure 1. Since Na_2_PdCl_4_ was essentially depleted from the reaction mixture at *t*=20 min, we stopped the synthesis at *t*=30 min to enable a direct comparison between the products prepared using different precursors. At *t*=30 min, the products obtained *via* one‐shot injection of Na_2_PdCl_4_ showed faster growth compared to that involving Pd(NH_3_)_4_(NO_3_)_2_. Due to the faster reduction, the concentration of the freshly‐produced Pd atoms exceeded the critical limit for homogeneous nucleation and the nuclei further grew into Pd particles of *ca*. 3 nm in size, in addition to their deposition on Ag nanocubes (Figure S4A). This observation is in agreement with the reduction kinetics summarized in Figure 1. By etching away the Ag template, Pd‐based nanoframes with a high coverage on side faces were obtained (Figure S4C), while the products synthesized with the same amount of Pd(NH_3_)_4_(NO_3_)_2_ barely showed any coverage on the side faces (Figure 4F). This result can be attributed to the faster reduction kinetics of Na_2_PdCl_4_, as well as the galvanic replacement reaction between the precursor and Ag template. With 0.2 mL of Na_2_PdCl_4_ added into the synthesis, galvanic replacement became dominant, leading to a disordered deposition of Pd atoms, and thus poorly‐defined Pd‐based nanoframes (Figure S4B and S4D). For the synthesis involving Na_2_PdCl_4_, the galvanic replacement has a positive reaction potential, indicating a spontaneous reaction as reported in a previous paper.^[41]^ In the case of Pd(NH_3_)_4_(NO_3_)_2_, however, the reaction potential for galvanic replacement is negative, implying that the reaction would become unfavorable due to the significant decrease in the reduction potential of Pd(NH_3_)_4_^2+^. With both slower reduction kinetics and exclusion of galvanic replacement, we achieved uniform deposition of Pd atoms on Ag nanocubes for the generation of well‐defined Pd‐based nanoframes with Pd(NH_3_)_4_(NO_3_)_2_.

**Figure 5:**
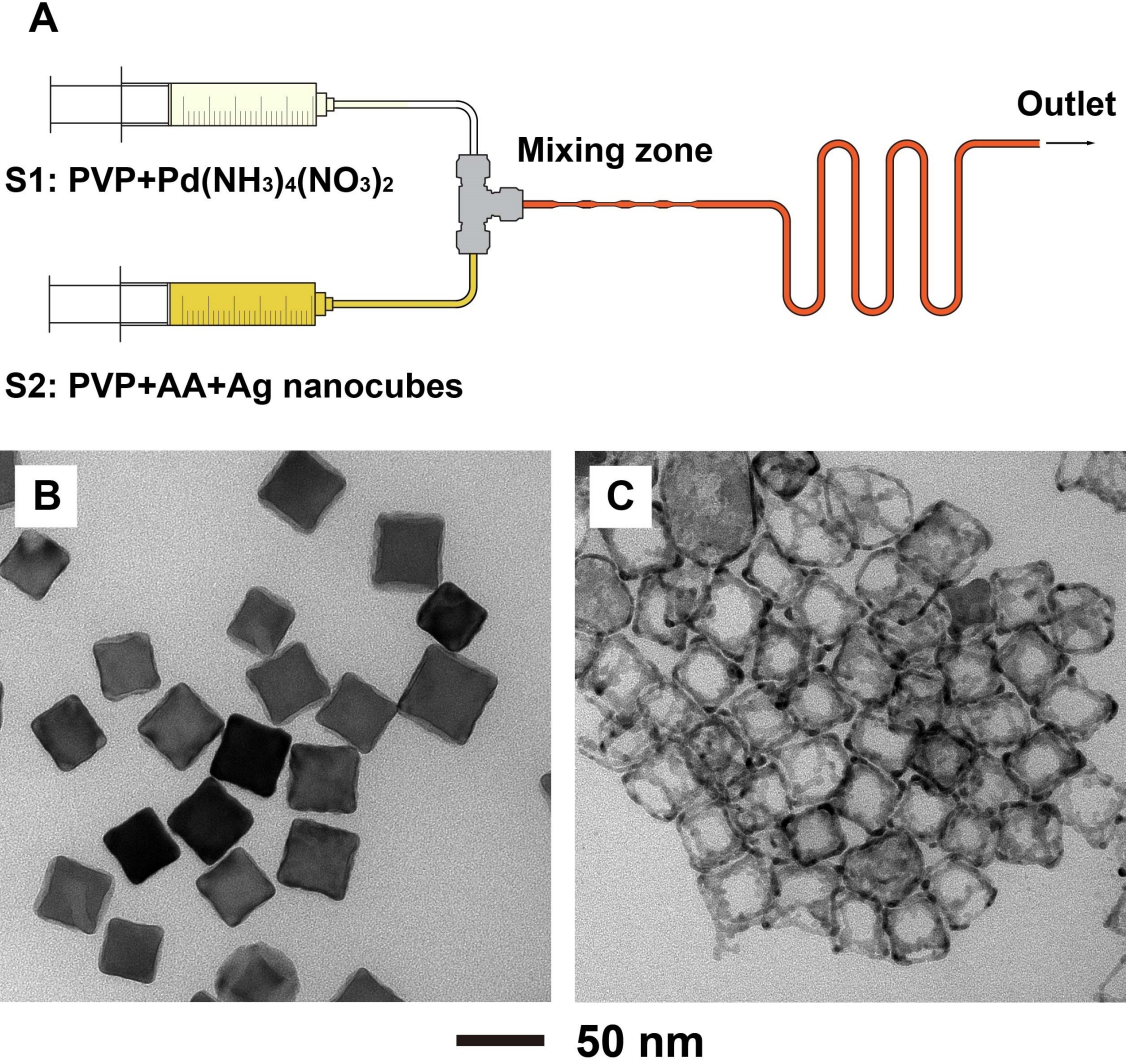
(A) Schematic illustration of the flow reactor used for the synthesis of (B) Ag@Pd core‐frame nanocubes. (C) TEM image of the resultant nanoframes obtained *via* H_2_O_2_ etching of Ag.

After the successful synthesis of Pd‐based nanoframes with Pd(NH_3_)_4_(NO_3_)_2_ in the setting of one‐shot injection, we further extended the synthesis from a batch reactor to a continuous flow reactor.[^42^, ^43^, ^44^] Figure 5A shows a schematic illustration of the homemade flow reactor. Two syringes, separately loaded with a mixture of PVP and the precursor and a mixture of PVP, AA, and Ag nanocubes, were mounted on two syringe pumps and set to an injection rate of 6 mL/min. The ratio between the reagents was set the same as the reagents used in the one‐shot synthesis involving 0.2 mL of precursor. The two reaction solutions were mixed through the use of a T connector and a mixing zone. The mixing zone contained tubes with a smaller inner diameter to create turbulence in the tube for achieving more uniform mixing in the flow. After all the reagents had been mixed in the tube, the pumps were turned off for a period of 30 min before collecting the final products. This modification allowed us to use a PTFE tube of only 0.5 m long for the proof‐of‐concept demonstration. Otherwise, a PTFE tube of 10 m in length had to be used to accommodate a reaction time of 30 min.

Using the flow reactor, Ag@Pd core‐frame nanocubes were produced with quality comparable to those prepared using one‐shot synthesis, together with good reproducibility (Figure 5B and C). The Pd‐based nanoframes were then obtained using the same etching method after the collection of Ag@Pd core‐frame nanocubes from the flow reactor. The Pd‐based nanoframes had more rigid edges and a little more coverage on the side faces compared to the product shown in Figure 2D, but were generally maintained as frames. This work demonstrated the feasibility of scaling up the synthesis of Pd‐based nanoframes without compromising the morphology of the products. It is also worth mentioning that the reaction in the flow reactor only took 30 minutes, only half of the duration used for a one‐shot synthesis. This could be attributed to the absence of oxidative etching in the PTFE tube when compared to the synthesis conducted in a vial. The oxidative etching could dissolve the Pd adatoms at sites with high surface energies and control their further deposition and diffusion. Without the involvement of oxidative etching, Pd atoms would be preferentially deposited on the edges and corners by following the order of surface energies, together with diffusion to the side faces when an excessive amount of the Pd precursor was reduced. The Pd‐based nanoframes synthesized in a flow reactor for 60 min were also collected for further investigation (Figure S5). At *t*=60 min, nanocages rather than nanoframes were obtained as the side faces were covered by a large amount of Pd atoms. However, the wall thickness of the nanocages was thinner than that of the ridges of the nanoframes due to the limited supply of Pd atoms determined by the amount of the precursor. This result offers evidence to support that the Pd atoms on side faces likely originated from the edges as a result of surface diffusion. Taken together, by controlling the amount of the precursor and/or the reaction time in the flow reactor, we could synthesize Ag@Pd nanocubes with quality comparable to the products prepared using one‐shot injection, thereby obtaining similar Pd‐based nanoframes after further etching.

## Conclusions

In summary, we have demonstrated the successful synthesis of Ag@Pd nanocubes *via* one‐shot injection of Pd(NH_3_)_4_(NO_3_)_2_ precursor into a mixture of Ag nanocubes, AA, and PVP at room temperature, and then obtained Pd‐based nanoframes by selectively etching away the Ag core with aqueous H_2_O_2_. The Pd(NH_3_)_4_(NO_3_)_2_ precursor exhibited slow reduction kinetics compared to PdCl_4_^2−^, preventing self‐nucleation while enabling spatially‐controlled deposition of Pd atoms on the Ag nanocubes. The lower reduction potential of Pd(NH_3_)_4_(NO_3_)_2_ also mitigated the galvanic replacement reaction, facilitating uniform deposition of Pd atoms. By gradually increasing the volume of the precursor introduced into the reaction system, the deposition of Pd atoms was controlled to occur on the edges and corners and then allowed to diffuse to the side faces of the Ag nanocubes. The growth pathway was also investigated, revealing that Pd atoms were initially deposited as small islands and then fused into rigid nanoframes over time. Finally, we demonstrated the feasibility of large‐scale synthesis in a continuous flow reactor, producing high‐quality Ag@Pd nanocubes and Pd‐based nanoframes comparable to those obtained through one‐shot synthesis in a batch reactor.

## Experimental Section

### Chemicals and Materials

Ethylene glycol (EG) was purchased from J. T. Baker. Silver trifluoroacetate (CF_3_COOAg, 99.99 %), poly(vinylpyrrolidone) with an average molecular weight of 29,000 (PVP‐29k) and 55,000 (PVP‐55k), sodium hydrosulfide hydrate (NaHS⋅xH_2_O), aqueous hydrochloric acid (37 % by weight), L‐Ascorbic acid (H_2_Asc, ACS reagent, ≥99 %), tetraamminepalladium(II) nitrate solution (Pd(NH_3_)_4_(NO_3_)_2_, 10 wt.% in H_2_O, 99.99 %), hydrogen peroxide solution (H_2_O_2_, containing inhibitor, 30 wt % in H_2_O, ACS reagent) were all purchased from Sigma‐Aldrich. Deionized (DI) water with a resistivity of 18.2 MΩ cm under ambient condition was used throughout the experiments.

### Synthesis of Ag Nanocubes

We followed a published protocol to prepare the Ag nanocubes with an average edge length of 35±3 nm.^[35]^ The Ag nanocubes were crushed out with acetone, collected by centrifugation, washed with water twice, and re‐dispersed in water at a concentration of 5×10^12^ particles/mL for further use.

### Synthesis of Ag@Pd Nanocubes and Pd‐based Nanoframes under One‐Shot Injection

In a standard synthesis, we introduced 2 mL of 1 mM PVP‐29k aqueous solution into a 23‐mL glass vial, followed by the addition of 0.5 mL of aqueous AA solution (100 mM) and 20 μL of the aqueous suspension of Ag nanocubes (1×10^11^ particles) under magnetic stirring at 600 rpm. Next, we injected different volumes of aqueous Pd(NH_3_)_4_(NO_3_)_2_ solution (0.2 mM) into the vial in one shot. After reaction for 1 h, we collected the solid products by centrifugation at 10,000 rcf for 15 min, washed with water three times, and then dispersed in water for further use. The etching of Ag from the Ag@Pd nanocubes was carried out by mixing the as‐obtained sample (1×10^10^ particles in total) with 1 mL of 3 % aqueous H_2_O_2_ at room temperature for 3 h. The resultant nanostructures were washed twice with water and then dispersed in water for TEM characterization. For the synthesis involving Na_2_PdCl_4_, the details remained the same, except for the substitution of Pd(NH_3_)_4_(NO_3_)_2_ with Na_2_PdCl_4_ and reduction of reaction time from 60 to 30 min.

### Quantitative Analysis of Reduction Kinetics

In a typical study, 0.2 mL of aliquot was sampled from the reaction mixture at different time points of a synthesis and quenched in an ice bath to prevent further reaction. The aliquot was then mixed with 0.8 mL of iced water, followed by centrifugation to leave behind the unreacted Pd precursor in the supernatant. The supernatant was then collected and diluted for elemental analysis by ICP‐MS.

### Synthesis of Ag@Pd Nanocubes and Pd‐based Nanoframes in a Flow Reactor

The flow reactor system was assembled from commercially available components, including two syringe pumps, PTFE tubes, and one polyetheretherketone (PEEK) T connector as shown in Figure 5A. During a standard synthesis, the flow reactor system was operated at flow rates of 6 mL/min for the aqueous solutions. These flow rates were found to be optimal for a better mixture of reagents and the synthesis of nanocrystals. After a certain volume of precursor and PVP, AA, and Ag nanocubes had been introduced into the exiting PTFE tube, we turned off the syringe pumps to extend the reaction time. For the synthesis of Ag@Pd nanocubes in the flow reactor system, S1 is loaded with a mixture of 0.4 mL of Pd(NH_3_)_4_(NO_3_)_2_ (0.2 mM) and 2.3 mL of PVP‐29k (1 mM), and S2 is loaded with a mixture of 1 ml of AA (100 mM) and 1.7 mL of PVP‐29k (1 mM). The final product was collected by centrifugation at 10000 rcf for 15 min and washed with DI water three times for TEM characterization and etching. Further etching process was the same as the one‐shot injection synthesis.

**Instrumentation and Characterizations**

We used an Eppendorf 5424 centrifuge to collect and wash all solid samples. TEM images were collected on a Hitachi HT7700 microscope operated at 120 kV. We performed high‐angle annular dark‐field scanning electron microscopy (HAADF‐STEM) imaging and EDS mapping on an FEI F30 TEM operated at 300 kV. We collected the XRD pattern of Pd‐based nanoframes using a Rigaku Miniflex X‐ray diffractometer with Cu Kα radiation (λ = 1.5418 Å). We also determined the elemental content in the samples and reaction solutions using a Thermo iCAP RQ ICP‐MS.

## Conflict of Interests

There are no conflicts to declare.

1

## Supporting information

As a service to our authors and readers, this journal provides supporting information supplied by the authors. Such materials are peer reviewed and may be re‐organized for online delivery, but are not copy‐edited or typeset. Technical support issues arising from supporting information (other than missing files) should be addressed to the authors.

Supporting Information

## Data Availability

The data that support the findings of this study are available from the corresponding author upon reasonable request.
