# Supplementary material for: Synthesis of Ag@Pd Nanocubes and Pd‐based Nanoframes via One‐Shot Injection of a Halide‐Free Precursor for Continuous Production in a Flow Reactor
Source: Chemistry. 2025 Mar 21;31(23):e202500201. doi: 10.1002/chem.202500201 (PMC12015395; doi:10.1002/chem.202500201)
Supplement: Supplementary file 1 — Supporting Information [file CHEM-31-e202500201-s001.pdf]

Chemistry–A European Journal

Supporting Information

Synthesis of Ag@Pd Nanocubes and Pd-based Nanoframes via One-Shot Injection of a Halide-Free Precursor for Continuous Production in a Flow Reactor

Hansong Yu, Jianlong He, Kei Kwan Li, Qijia Huang, Yong Ding, and Younan Xia*

Supporting Information

Synthesis of Ag@Pd Nanocubes and Pd-Based Nanoframes *via* One-shot Injection of a Halide-free Precursor for Continuous Production in a Flow Reactor

Hansong Yu, Jianlong He, Kei Kwan Li, Qijia Huang, Yong Ding, Younan Xia*

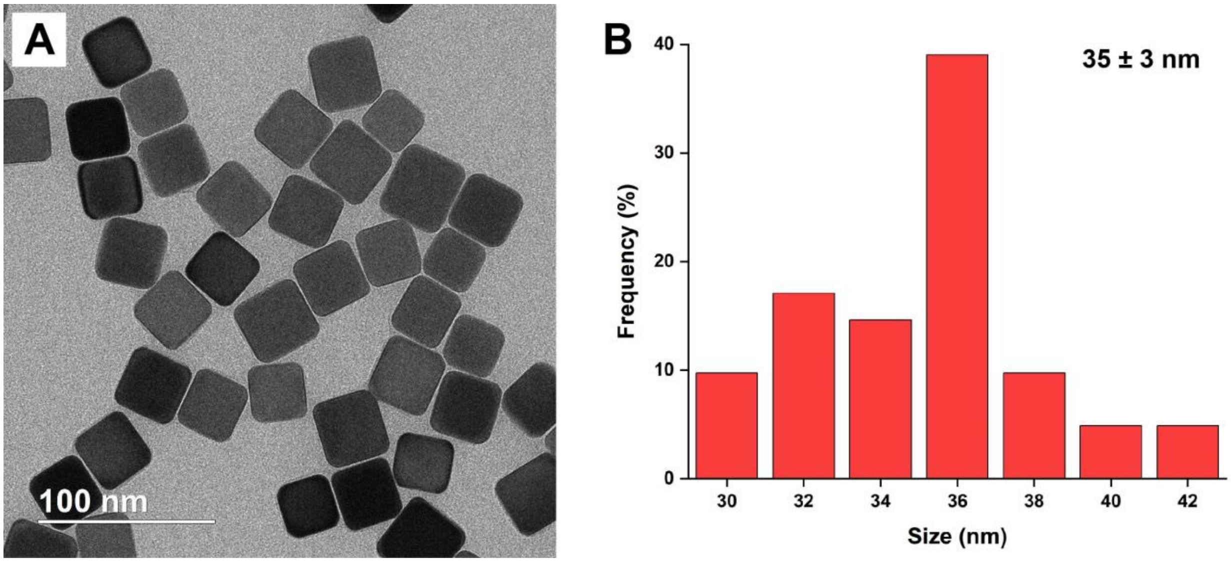

Figure S1. (A) TEM image of the Ag nanocubes used in the present study and (B) a plot of their size (edge length) distribution.

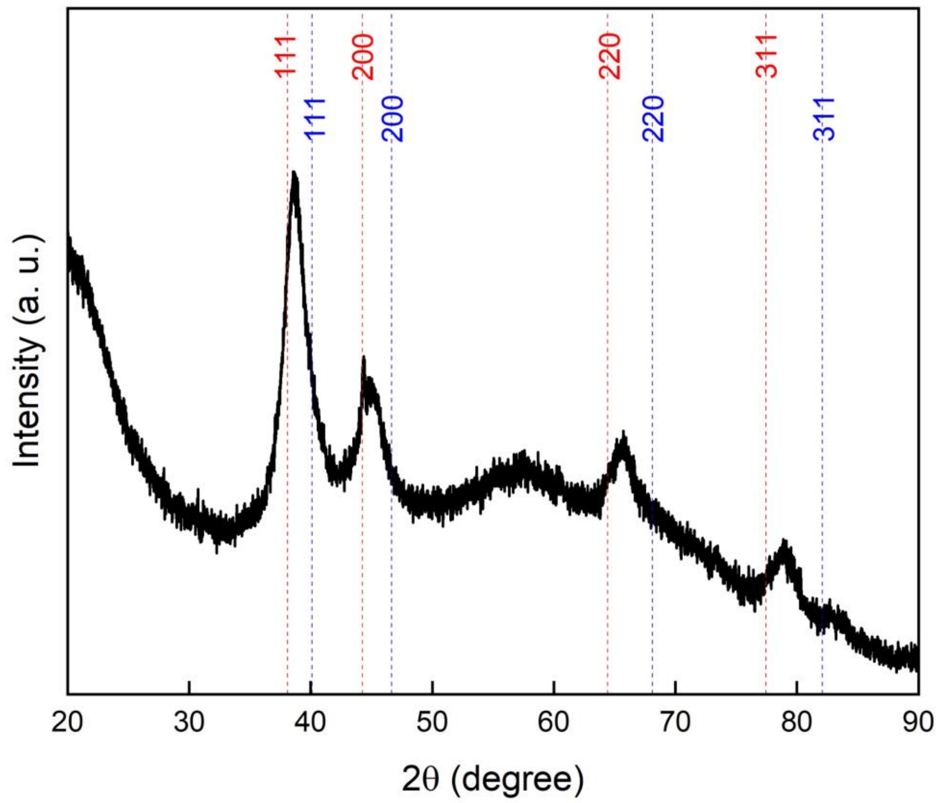

Figure S2. XRD patterns of the Pd-based nanoframes prepared with 0.3 mL of $Pd(NH_3)_4(NO_3)_2$ (0.1 mM). The red and blue dashed lines correspond to the characteristic peaks of Ag (JCPDS No. 04-0783) and Pd (JCPDS No. 46-1043), respectively.

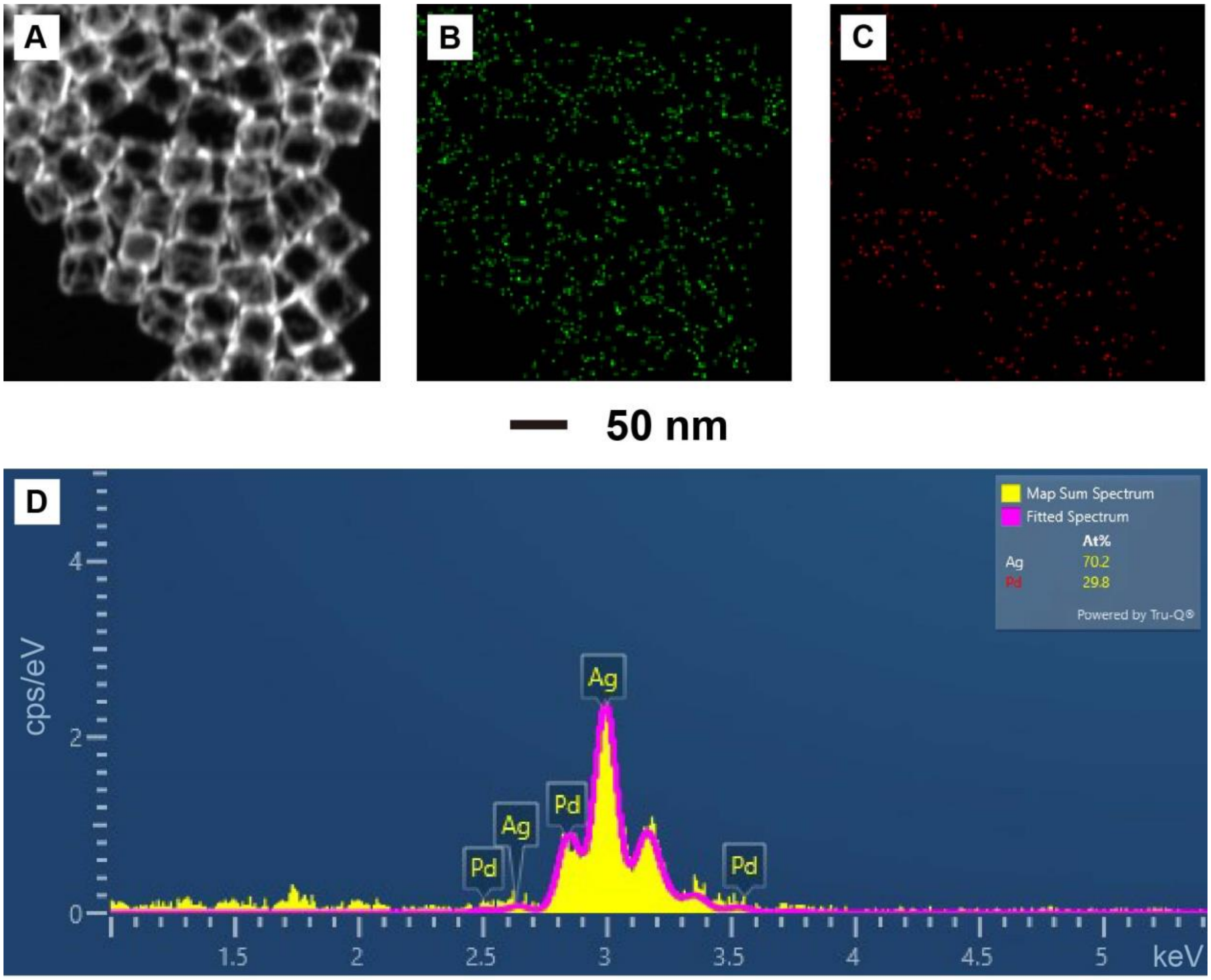

Figure S3. (A) HAADF-STEM image of Pd-based nanoframes derived from the Ag@Pd core-frame nanocubes prepared with 0.3 mL of $Pd(NH_3)_4(NO_3)_2$ solution (0.1 mM). (B, C) Elemental mapping of Ag (green) and Pd (red) in (A), respectively. (D) Corresponding EDS spectrum of (B) and (C).

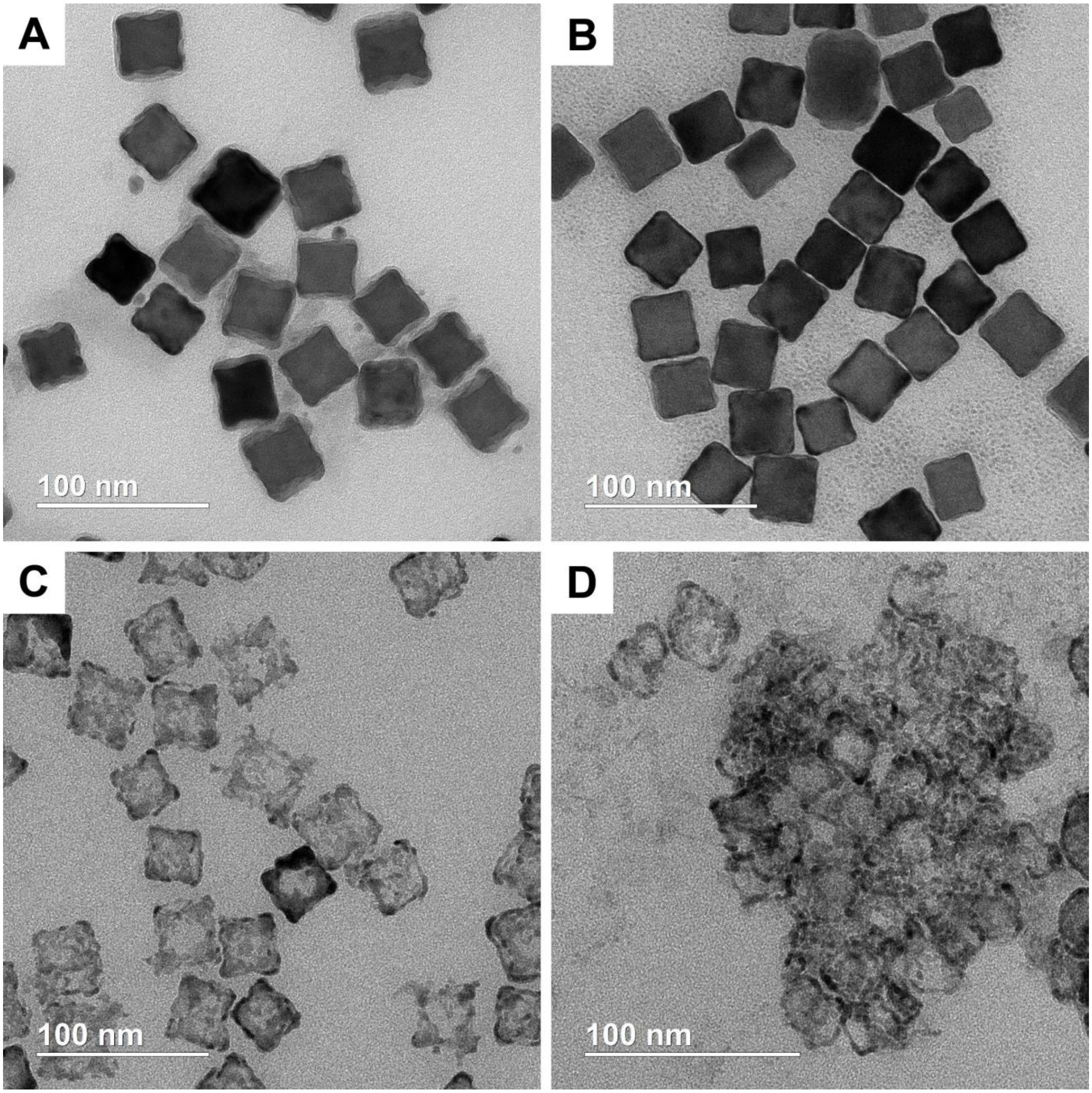

Figure S4. TEM images of the Ag@Pd nanocubes synthesized through one-shot injection of (A) 0.3 and (B) 0.2 mL of Na_2PdCl_4 (0.2 mM), respectively. (C, D) TEM images of the resultant nanostructures obtained after etching the corresponding sample of Ag@Pd nanocubes with aqueous H_2O_2.

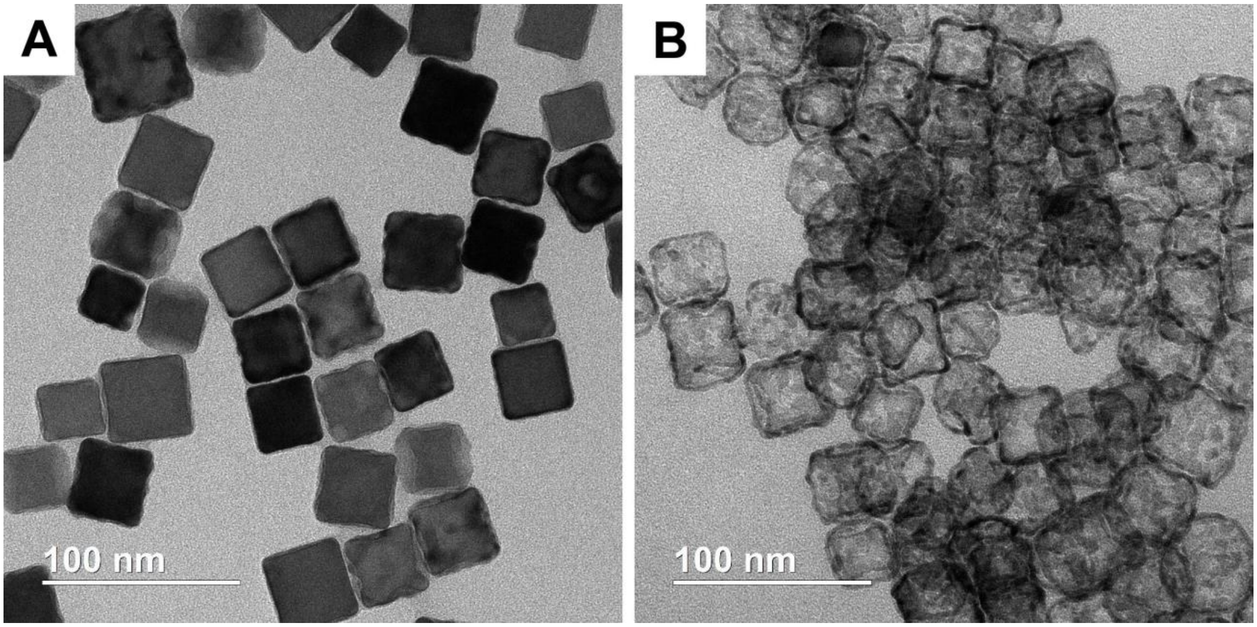

Figure S5. (A) TEM images of the Ag@Pd nanocubes synthesized in a flow reactor for 60 min with a 3 to 1 ratio, equivalent to that in the synthesis involving one-shot injection of 0.2 mL of the Pd precursor. (B) TEM images of the nanostructures obtained after etching the corresponding sample of Ag@Pd nanocubes with aqueous H_2O_2.
